# Advanced materials design based on waste wood and bark

**DOI:** 10.1098/rsta.2020.0345

**Published:** 2021-09-20

**Authors:** Charlett Wenig, John W. C. Dunlop, Johanna Hehemeyer-Cürten, Friedrich J. Reppe, Nils Horbelt, Karin Krauthausen, Peter Fratzl, Michaela Eder

**Affiliations:** ^1^ Department of Biomaterials, Max Planck Institute of Colloids and Interfaces, Am Mühlenberg 1, 14476 Potsdam, Germany; ^2^ Department of the Chemistry and Physics of Materials, Paris Lodron University of Salzburg, Morphophysics Group, Salzburg, Austria; ^3^ Cluster of Excellence ‘Matters of Activity. Image Space Material’ at Humboldt Universität zu Berlin, Berlin, Germany

**Keywords:** cellulose, fibre, waste material, weaving, forest residues

## Abstract

Trees belong to the largest living organisms on Earth and plants in general are one of our main renewable resources. Wood as a material has been used since the beginning of humankind. Today, forestry still provides raw materials for a variety of applications, for example in the building industry, in paper manufacturing and for various wood products. However, many parts of the tree, such as reaction wood, branches and bark are often discarded as forestry residues and waste wood, used as additives in composite materials or burned for energy production. More advanced uses of bark include the extraction of chemical substances for glues, food additives or healthcare, as well as the transformation to advanced carbon materials. Here, we argue that a proper understanding of the internal fibrous structure and the resulting mechanical behaviour of these forest residues allows for the design of materials with greatly varying properties and applications. We show that simple and cheap treatments can give tree bark a leather-like appearance that can be used for the construction of shelters and even the fabrication of woven textiles.

This article is part of the theme issue ‘Bio-derived and bioinspired sustainable advanced materials for emerging technologies (part 1)’.

## 1. Introduction

**Figure 1.**
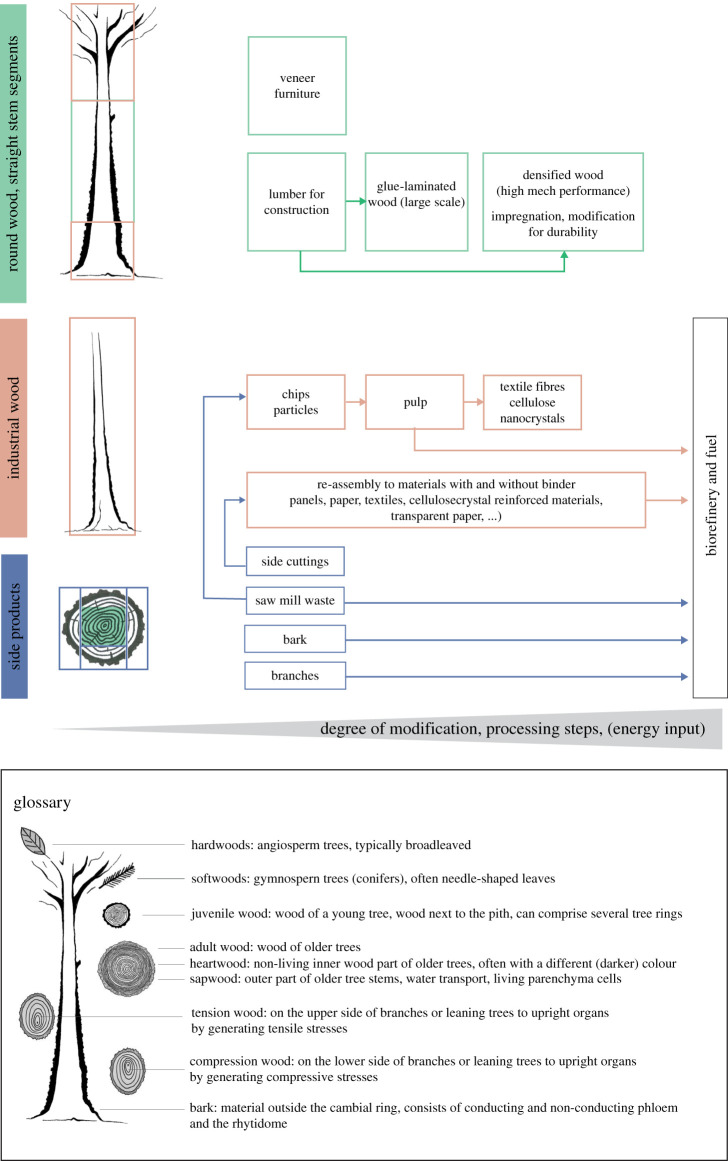
Tree material use. The trees on the left side illustrate an old tree (top) and a young tree (below). The disc of the stem illustrates proportions of wood and bark and side cuttings. The chart of tree material use shows possible applications as well as tree waste (parts of trees) which go directly into the biorefinery or are used as fuel. The glossary at the bottom describes different parts of the tree with hardwood reaction wood on the left and softwood reaction wood on the right (excentric cross-sections). (Online version in colour.)

Cellulose is the most abundant polymer on Earth and trees are a major part of this biomass. Modern forestry technologies concentrate on harvesting stems as their most valuable part, leaving reaction wood, branches and bark as forest residues and waste wood. The main reason is that the ultrastructure of the material outside the main part of the wood is more variable and, therefore, more costly to directly put into use. Some typical usages of trees are shown in figure 1.

Plants grow in an adaptive way depending on the local environmental conditions, such as the land slope, side winds or other mechanical challenges. Trees develop reaction wood in some parts of the stem as well as in branches to compensate for loads that change over time [1]. The resulting properties of the wood material are, therefore, more variable in these regions and more difficult to put into commercial use. In this paper, we argue that the structure of these materials is by no means random but follows known principles of adaptation [2], so that a knowledge-based advanced use is not excluded. We firstly give an overview into what is known about structure–properties–function relationships in wood and highlight that this is only part of the total usable biomass of a tree (§§2 and 3). Through the help of one specific example, namely bark, we discuss a process of alternative waste material use as well as difficulties that arise from a lack of basic knowledge of material properties. Currently, the cellulosic material bark is mostly used for energy production or as additive in wood-based composites [3]. More advanced uses include the extraction of adhesives [4], or of compounds relevant for the food industry or healthcare [5,6]. Carbonization of bark can even be used to obtain carbon nanosheets for supercapacitor electrodes [7]. In this paper, we show that simple and cost-effective treatments of bark can confer it very different mechanical properties. In particular, bark can be transformed into a material with leather-like appearance that can be manufactured into woven textiles (§4).

## 2. Building blocks, structural patterns and properties of materials from trees

Plants make up by far the largest amount (approx. 80%) of biomass on Earth [8]. Their sizes range from small unicellular organisms such as green algae to the heaviest known organism, the giant aspen clone ‘Pando’ [9,10]. Parts of the living organism, the plant, can be seen as and are used as materials, with the proportions of useful material increasing with growth. But what is a ‘plant material’? Typically, plant materials are taken to be materials derived from or extracted from plants. These plant materials can include seeds/husks (e.g. cotton, kapok, coir), stems (wood, flax, hemp, bamboo, sugar cane), bark (cedar bark, cork of oak, birch bark), leaf fibres (sisal, agave) or roots (spruce root, willow root, cedar root), waxes and resins. We feel that it is useful to broaden the concept of plant materials to include them as being part of the living plant before harvest, as they serve the plant's biological functions as support, transport vessel and even actuation or force generation. This change may help us to better understand plant materials in the context within which they were formed and grown.

To expand this viewpoint, we first look at the cell, the smallest living building block. One characteristic of plant cells is the presence of a cell wall, located outside the plasma membrane. The plasma membrane contains protein complexes, called cellulose synthase units (CESAs), that move within the membrane to produce and secrete stiff, strong and long cellulose microfibrils on the outside of the cell. Hemicelluloses and pectins are synthesized by enzymes localized in the Golgi and transported by the endomembrane system towards the plasma membrane [11]. The spatial arrangement of the cell wall polymers, together with growth directions of the tissues and external restrictions of the neighbouring cells, determines cell shapes and geometries [12]. After cell growth has ceased, many cells form secondary cell walls—composites of cellulose, hemicelluloses and lignin, a complex polymer consisting of phenolic precursors. Compared to the primary cell walls, secondary cell walls are much thicker and the cellulose fibrils are arranged mainly parallel to each other [13]—at least in fibrous cells. Many cells with secondary cell walls die shortly after formation allowing them to provide a function to the plant in water transport and mechanical stability. Agglomerations of dead cells, such as large proportions of wood or bark, can thus be seen as functional material even though they are still essential parts of aliving organism.

**Figure 2.**
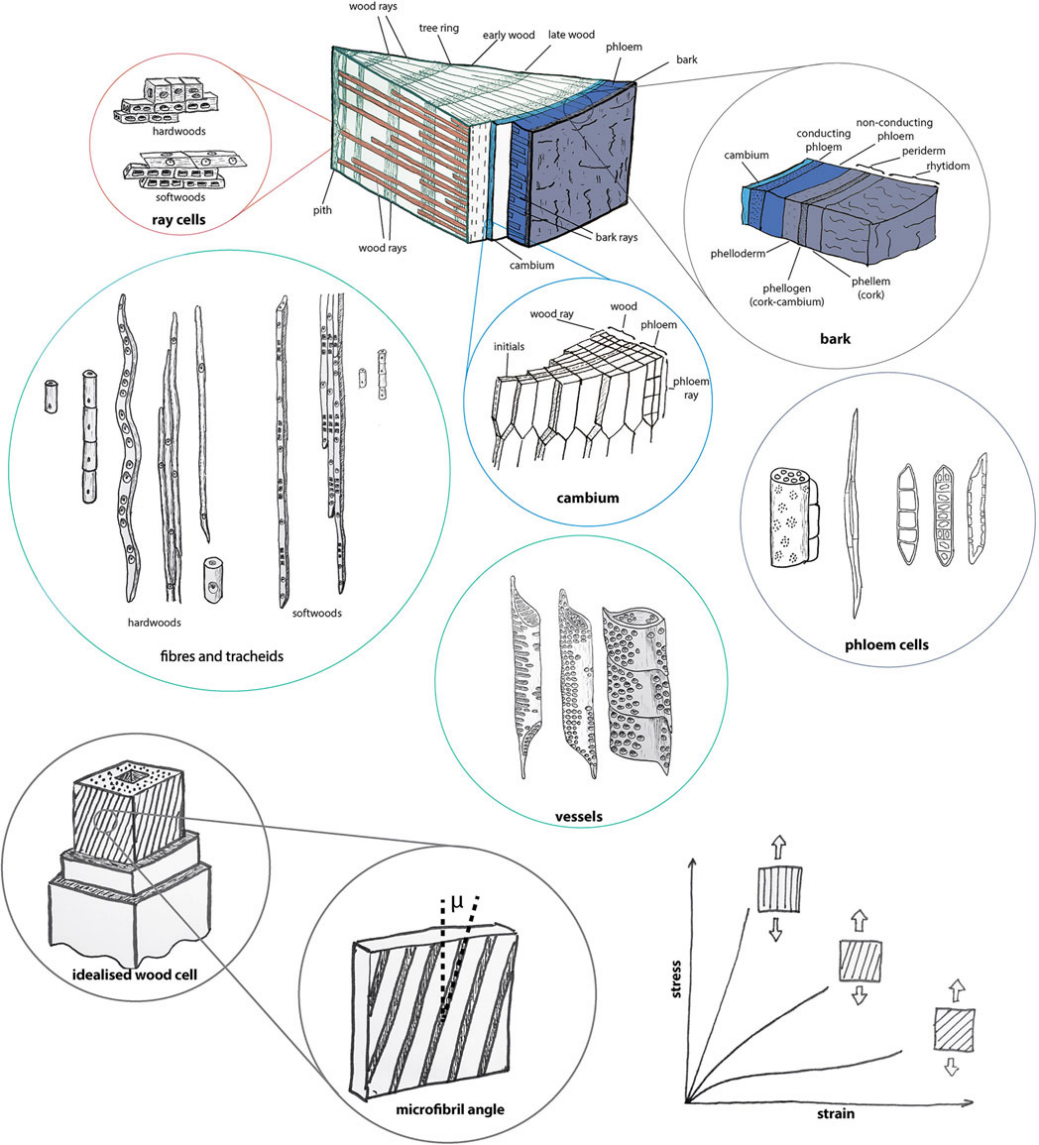
Schematic of the stem of a tree showing different tissue and cell types. The ‘piece of cake’ shows a macroscopic segment (several centimetres) of a tree stem from the pith to the outer bark with its different tissue types. From the outside: bark (blue and grey), cambium (turquoise), wood (green) with ray cells (orange). Coloured circles show the cell types and geometries of these tissues at higher magnifications (cell diameters between 5 and 40 µm, lengths up to 3 mm). Grey circles on the bottom show secondary cell wall structure with parallel cellulose orientation (microfibril diameters approx. 30 nm) and the diagram illustrates the effect of the cellulose orientation on the mechanical properties. Drawings partly inspired by the figures in [14–16]. (Online version in colour.)

Trees, due to their abundance and large size, are of particular interest in the context of plant materials. Despite their size, large proportions of trees—wood fibres, tracheids, vessels and the rhytidome (figure 2)—consist of dead cells with secondary cell walls. The cellulose microfibrils, indicated as black lines in figure 2 (bottom circles and diagram), are the essential building blocks of the cell walls. With a very high stiffness (greater than 120 GPa) and strength (7 GPa) [13,17], they are reinforcing structural elements, akin to the stiff glass fibres in glass-fibre-reinforced polymers. Their spatial arrangement with respect to loading directions (diagram in figure 2) determines mechanical [18] but also swelling properties [19,20]. Cellulose orientation is defined by the microfibril angle (MFA), given by the angle of the majority of cellulose microfibrils with respect to the longitudinal axis (figure 2). The MFA plays an important role, especially in fibrous cells, which are abundant in wood and to a lesser extent in bark. These cells can also be found in fruits and other seed containers. Cells with a small MFA are stiff and strong in tension, while cells with a large MFA are less stiff and strong [18]. For other cells, such as isodiametric cells, pavement cells or three-dimensional puzzle cells, relatively little is known about the cellulose orientation and its role on the mechanics of cells.

**Figure 3.**
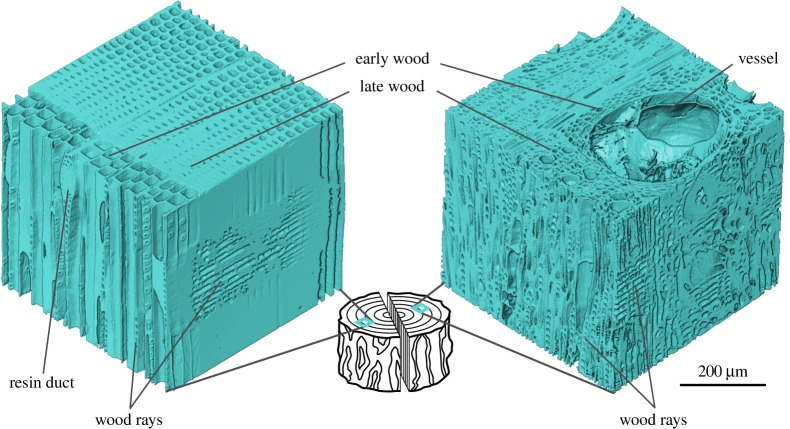
µCT scans of spruce and oak wood. The CT scan of spruce wood (left) shows the distinct growth ring boarder with thin-walled early wood cells for efficient water transport and thick-walled latewood cells contributing more to the mechanical stability. The wood rays are narrow and ray cells are visible on the radial plane. Radial resin duct on the tangential plane. The µCT scan of oak shows the higher complexity of angiosperm wood with vessels, tracheids, libriform fibres, narrow and very broad rays. (Online version in colour.)

Wood cells are formed by the cambium at the periphery of the stem (figure 2). The wood cells (figure 2) are arranged in specific patterns (figure 3) that provide functionality, such as water and nutrient transport, storage and mechanical stability. This requires not only a detailed physico-chemical characterization but also needs to be understood in the context of the sessile plant's environmental conditions and the needs of the plant. This can be illustrated by the flexibility of a young tree, which bends rather than breaks upon wind or animal loading. Young trees consist to a large extent of wood with a low density and a large MFA. As the tree becomes larger, stiffness is needed to sufficiently support the crown. The absence of tissue remodelling processes in trees means that they can only adapt to changing conditions by growth—by adding new cells with various geometries and cell wall structures in regions of the tree where needed. To support the increasingly heavier crown, trees grow the newer layers of wood with denser cells, and with lower MFAs. Once a tissue is formed, the microstructural features such as density and MFAs are imprinted and stored within the material for the life of the tree and beyond. Hence, a simple, straight stem consists of wood with a wide variety of properties. The inner material remains less dense with the larger MFA and the outer material being denser with smaller MFAs. Woven within and between the fibres, vessels and tracheids are the ray cells (figure 3), which can provide increased radial strength [21]. The dead material in the centre of the tree can still be modified by the tree long after the cells have formed, with numerous tree species forming heartwood. After the sapwood tracheids stop water transport and the parenchyma cells die, extractives are incorporated into the cell walls to increase natural durability [22]. This heartwood formation is a one-way process, leading to permanent changes in local properties. Towards the top of the tree are stem segments of juvenile wood without heartwood formation. Since trees are bound to a location, they also need to be able to react to changing loading conditions. Tilted stems, but also branches, form reaction wood. In the case of softwoods, compression wood is formed on the lower side to push the stem or branch by the generation of compressive stresses. Hardwoods form tension wood on the upper side and pull the organs in the desired direction [23]. The process of stress generation is based on structural differences at the cell wall, cell and tissue level [24,25], resulting in altered chemical and mechanical properties compared to ‘normal wood’. The ability of plants to move is not restricted to stems or branches. Seed structures especially are known to perform complex motions; however, due to their limited availability and small size, these structures are of little interest as a plant-based raw material. These materials rather serve as bioinspiration, for example for self-actuating (façade) elements [26–28] and provide a fertile research area for the development of bioinspired self-moving materials [29].

Besides wood, bark is also available on a large scale and in huge quantities; however, much less is known about the properties of bark than about wood. Recent publications partly address this missing information giving comprehensive information about bark anatomy of various species [30,31], macroscopic bark structure [31] and chemical composition [32,33]. Bark is the boundary of a tree stem to its environment and the term bark contains all the tissues from the cambium towards the outside (e.g. [14], figure 2). As the cambium synthesizes xylem cells (wood) towards the inside, it synthesizes to a lesser extent secondary phloem cells (sieve cells and tubes and bark ray cells, figure 2) towards the outside. The phloem consists of the inner conducting and the non-conducting phloem, which is located a bit further outside (figure 2). Even though the non-conducting phloem loses its conductive capacity after being formed, storage functions and meristematic potential remain. The outermost layer of a young tree seedling is the epidermis, which is not able to grow sufficiently to serve as a permanently protecting layer. At some stage, the epidermis ruptures and is replaced by the periderm, a secondary protective covering. The periderm consists of phellogen or cork cambium, a meristematic tissue, which produces the watertight phellem or cork towards the outside and the periderm towards the inside. The periderm separates non-functional phloem from the living inner part of the stem towards the outside, which forms the rhytidome. The location of the phellogen(s) in the plant plays an important role for the bark structure.

The main functions of bark are water and nutrient transport and storage. The outer bark protects the tree from external challenges such as frost, heat, fire or microbes. Owing to its peripheral location on the stem and hence its large second moment of area, bark also contributes to the mechanical integrity of tree stems and twigs, despite its lower modulus compared to wood [34]. Similar to other parts of the tree such as roots or leaves, bark is in permanent interaction with the environment and shaped by the environment and ‘bark-inhabitants’. It provides ‘housing’ for numerous other organisms: fungi, lichens, mosses, plants but also animals such as insects and birds. It is, therefore, not surprising that its properties are highly variable—not only between different tree species but also within species and even within a single tree.

## 3. Trees as raw material for applications: degrees of modification

We have seen above that tree stems and branches display a wide variety of material properties and functions. While bark and softwood branches are typically only used for low-value applications and as an energy source, they appear to be understudied compared to stem wood, which is often used for advanced applications (figure 1). Wood is one of the oldest useful materials of mankind: knowledge of the properties of various woods is documented already for the Neolithic period, i.e. 5000 years ago [35]. This ancient tradition is still contained in the etymology of the term, insofar as the ancient Greek term ὕ*λη* (*hyle*, Engl.: *matter/material*) or in Latin translation *materia* and *materies* primarily meant *wood*, both the forest and the material (timber) that was used for the construction of houses, ships and as ship masts [36]. In this respect, wood stands in as an ‘archetype’ for both the general concept of matter and for that of functional material [37]. Practical access to the wood as material ranges from the mythical ‘primitive hut’ referred to in architectural theory since Vitruvius (first century BC) [38] to modern timber building systems of prefabricated modules such as the ‘Packaged House System’ by Konrad Wachsmann and Walter Gropius (1940s), which could ‘grow’ additively in all directions, i.e. be extended. What is changing, however, is the way the material is handled: while it was initially an object of selection due to its complex structure and consequent properties (hygroscopicity, elasticity, inhomogeneity), in the twentieth century it was often used in a decontextualized way, i.e. homogenized into a passive building material (e.g. medium density fibre board) by crushing and in combination with resistant adhesives [35]. Only recently have attempts been made to transfer the structural potential of wood (its ability to react to environmental conditions) into constructions, for example in the experimental architectural projects of Menges & Reichert [26].

Natural, unmodified wood is again a material with highly desirable properties. Not only is it aesthetically pleasing, with applications in furniture making, and the production of veneers (a semi-finished product), it has a low thermal conductivity and can act as an insulator. Wood's high mechanical performance means that it is used as a structural material for constructions and buildings, especially when loaded along the fibre direction. In this direction, the stiffness is approximately five times higher than across the fibre direction due to the presence of the weaker lignin-rich middle lamellae between the longitudinally oriented cells. For applications requiring a minimal mass for a given performance, such as beams in construction, wood outperforms most synthetic materials and at much lower cost [39,40].

The desire to develop bio-based high strength and stiffness materials, which exceed the above-described mechanical properties, led to recent developments of so-called densified wood [41,42]. Partly delignified [42] or fully delignified [41] wood pieces are compressed until collapse of the fibres. During the process, the fibre orientation and the MFA remain and the material reaches a density of approximately 1.3 g cm^−3^. This densification leads to a drastic increase in tensile strength of up to 548 MPa [42] for a bulk material at the centimetre-scale which is almost as high as the ultimate stresses observed for wood cells (µm-scale) when tested in tension [43]. The described processes homogenize density and by this they reduce the biological variability of the material dramatically. This has the potential to use wood-based materials for applications which require reliable high mechanical performance—under consideration of the fibrous structure and the inherent directionality. Besides approaches to improve mechanical properties, numerous modification methods for a higher dimensional stability, durability, etc., are researched and applied (e.g. [44]). In summary, both natural wood and modified wood with excellent mechanical properties and comparably low biological variability are retrieved from mature stems, ideally having a low curvature of the tree rings and a small MFA.

However, not all parts of trees can be used for the described ‘high value applications' due to their geometries or material properties. The side cuttings of the sawn tree stems and the upper parts of trees with low diameters, low densities and larger MFAs are typically classified as industrial wood (figure 1). Industrial wood is reduced to smaller pieces ranging from strands to small chips and re-assembled to different types of panels (e.g. OSB, particle board). A considerable amount of industrial wood is raw material for making pulp, an energy and chemistry-intensive process for further disintegration involving the removal of lignin. The quality of pulp depends on the applied process (e.g. sodium sulfate, sodium sulfite process) but also on the raw material. To give an example, softwood pulp with much longer fibres than hardwood pulp is more suited for high-strength paper as used for packaging. Besides being the raw material for fibre-boards or paper, pulp is the starting material for wood-based textile fibre production (e.g. viscose and lyocell) but also for the production of cellulose nanocrystals (CNCs), which are used and researched for biomedical devices, nanocomposites, as emulsions, gels and foams [45] and for optical materials due to their ability to self-assemble [46].

The given examples illustrate the wide variety of how wood is currently used. The structure and composition of the raw material as well as the final product is essential for the performance, and the geometric element ‘fibre’, found at various length scales (cellulose fibre at the molecular level and fibre at the microscopic level), is the key parameter for the material properties.

**Figure 4.**
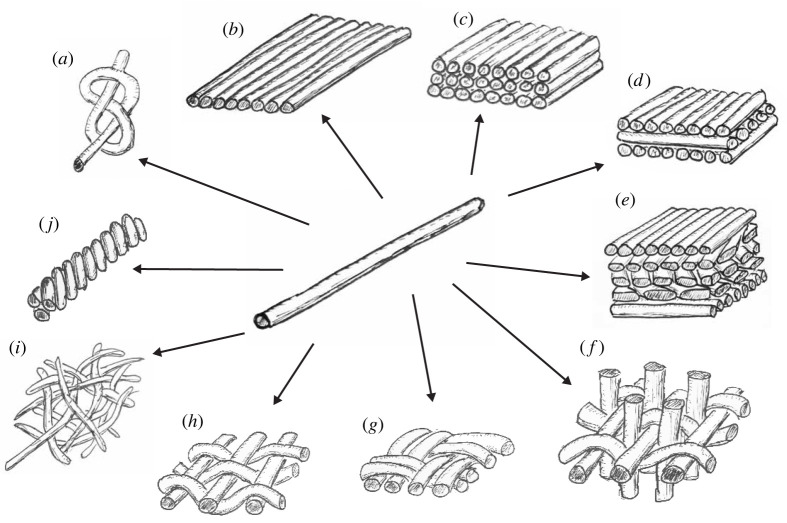
How single anisotropic fibres can be built up into macroscopic materials with new properties. Fibres can be knotted (*a*) arranged in layers (*b*) that can be stacked in different arrangements (*c*–*e*) giving anisotropic or almost isotropic properties. Fibres can be twisted (*j*), randomly arranged (*i*) or woven in two, (*g*,*h*) or three dimensions (*f*).

Many structural materials in biology have fibrous building blocks like the cellulose in plants, with collagen silks and chitin being typical examples [47]. The fibrous nature of these components and their inherent anisotropy mean that the ‘architecture’ or the spatial arrangement of these fibres at different length scales is a fundamental parameter in controlling the properties of the resultant tissue. For fibres, there are several ‘length-scale independent’ ways to construct a material (figure 4). Isotropic material properties can be achieved by stacking parallel oriented layers of fibres on top of each other with alternating twists as is well known in plywood [48]. Such features can be seen in many biological examples (osteons in bone, insect cuticle, plant cell wall) and are thought to improve toughness [49–52]. Twisted fibres (such as in the use of fibres in making cables and ropes) can allow for the transmission of tensile loads between a large number of shorter fibres, retaining transverse flexibility [53]. Weaving or the alternating crossing of fibres under and over each other can create two-dimensional fabrics, with the weave pattern controlling properties in particular directions [54]. In principle, weaving can also be done in three dimensions with fibres interlocking creating functionality in multiple directions [55,56], in some way reminiscent of the interwoven wood cells (rays and fibres) seen in figure 3. Random patterning (such as is found in fibre arrangements in paper manufacture, or in the production of filters or felts) can give uniform properties in the plane. These general thoughts on how fibres can be packed to achieve different functionalities can be seen at multiple length scales going from the molecular up to the macroscopic scale of a rope or plywood structure. Although the link between macroscopic properties and the underlying architecture of fibrous materials is understood, the biological processes that are responsible for the assembly of the fibres are still unclear. As a consequence, many research groups are currently working to try to understand how ‘fibrous patterning’ works in living materials such as wood, bone and cuticle. It is possible that part of the arrangement process is spontaneous, rather like the spontaneous arrangement of liquid crystals, or alignment is controlled through active processes of growth operating in geometric confinement.

Even though being fibrous materials, not all parts of the tree fit nowadays requirements to be used as material. Considerable amounts of branches and most barks are currently only used as energy wood or as fuel without any other previous application. We assume that one of the reasons is that the material properties within a small volume (e.g. a branch) can change and vary enormously. Branches grow eccentrically, are smaller in diameter with a large curvature of the growth rings and finally the chemical composition and MFA is heterogeneous over a cross-section. Consequently, the material behaviour is difficult to predict and only limited information about branch structures and properties for different tree species is available. If understood, branches may become interesting raw materials for self-moving and self-shaping structures due to their complex swelling and shrinkage behaviour.

While wood is valuable enough to be stored until dry, bark—with the exceptions of the cork of oak trees and some barks with valuable substances for extraction—is often seen as an even lower value material and frequently burnt in the wet condition to free storage capacities at the saw mills. In the light of the limited availability of resources, it makes sense to add value to bark produced in large volumes as a by-product of the wood industry. And in fact bark was used for a lot of purposes in different cultures. The finding of an approximately 8000 year old bark cloth beater in southern China is one of the earliest pieces of evidence for bark cloth making [57]. Early bark cloth is non-woven and made by beating the plant fibres after retrieving the inner bark. With human migration, the knowledge as well as the plants (e.g. paper mulberry) expanded to various regions [58,59]. Bark cloth is still made in islands of the Oceania as ‘tapa’ as well as in Central America and Uganda [60]. However, the inner bark material was also spun into cord or yarn and used for tying and knotting. This bast-like material could be knitted, woven and braided into two- and three-dimensional objects—bags knitted from bark yarn have been found in Australia, for example, and mats woven and braided from birch bark (from useful underlays to coverlets diligently worked with quills with feathers), boxes (from salt vessels to bags) and useful helpers such as the pot scourer (a ball made from rolled-up strips of bark) but also bark scrolls with drawings on it have been found in the Finnish/Russian region of Karelia.^1^ The objects from Karelia show that the resistance and durability of birch bark material in combination with its pliability made it possible to weave and braid the bark after it was cut into narrow strips. The advantage of the inner bark material consists in the fact that after processing (watering and beating), it behaves as a kind of *solides souples* (Engl.: *soft solids*) and in some aspects as an analogue to paper [61]. This is confirmed by the use of birch bark for writings such as in the Gandhāran Buddhist manuscripts that date from approximately the first century CE and are believed to have been created in Afghanistan.^2^ However, the outer bark was known as a useful material and even as a construction material: for hundreds of years, North American Indians used birch bark for their canoes [62], and in Austria an old tradition is remembered where the loggers used bark as construction material for temporary huts in the forest (e.g. bark huts in Hof close to Salzburg or in the Salzburger Freilichtmuseum). Current bark use is limited to agricultural and gardening applications to improve soil quality, as an energy source or as a feedstock for biorefinery to extract substances such as betulin (e.g. [63]), tannins (e.g. [64]) or lignin (e.g. [65]). Owing to the use of bark as biorefinery feedstock comprehensive information about extractives, tannins, polysaccarides, lignin and suberin is available [32,33]. Approaches to use bark in panel production started already in the 1960s, especially for particle boards [66,67]. More recently, larger bark particles (chips) were pressed to panels by adding binders and their potential to be used for insulation purposes was explored [68].

The examples show that tree material with predictable properties such as stem wood is typically used in an almost unmodified way. With increasing biological variability as observed in, for example, branches, the degrees of disintegration and modification of the raw material for the desired applications increase, involving environmental footprints which are possibly not neglectable and require further research effort to fully quantify them. For example, it was shown recently that for CNCs, the environmental impact by water and energy consumption, carbon footprint and missing biorefinery concepts were unclear with hardly any comprehensive life cycle assessments available products made of these renewable raw materials [45].

## 4. Bark: skin of trees and potential use as textiles

As described in the previous section, branches and barks of trees are mainly seen as low value or even waste material. This section discusses alternative interdisciplinary approaches to add value to such materials using the example of bark. The use of bark as a textile is not new, with many historical examples from around the world. What is new, however, is the scale of bark produced as a waste material from the wood industry. In the last century, this has been seen as a problem to be disposed of and not as a resource.

Bark, in terms of its composition and structure, is at least as diverse as wood and differences between species may display different environmental challenges in different ecosystems. Trees in cold regions need to protect the living cells against intracellular ice formation. In very cold regions, this protection is mainly biochemical [69,70] while a physically protective role of the bark is possible in regions with more random and shorter frost events, as seen in savannahs [71]. Trees growing in fire-prone regions often possess fire-resistant barks which protect the underlying meristematic tissues against high temperatures [72], at the same time protection against mechanical damage may play an important role, as recently shown for giant sequoias growing in regions with regular rockfalls [73].

**Figure 5.**
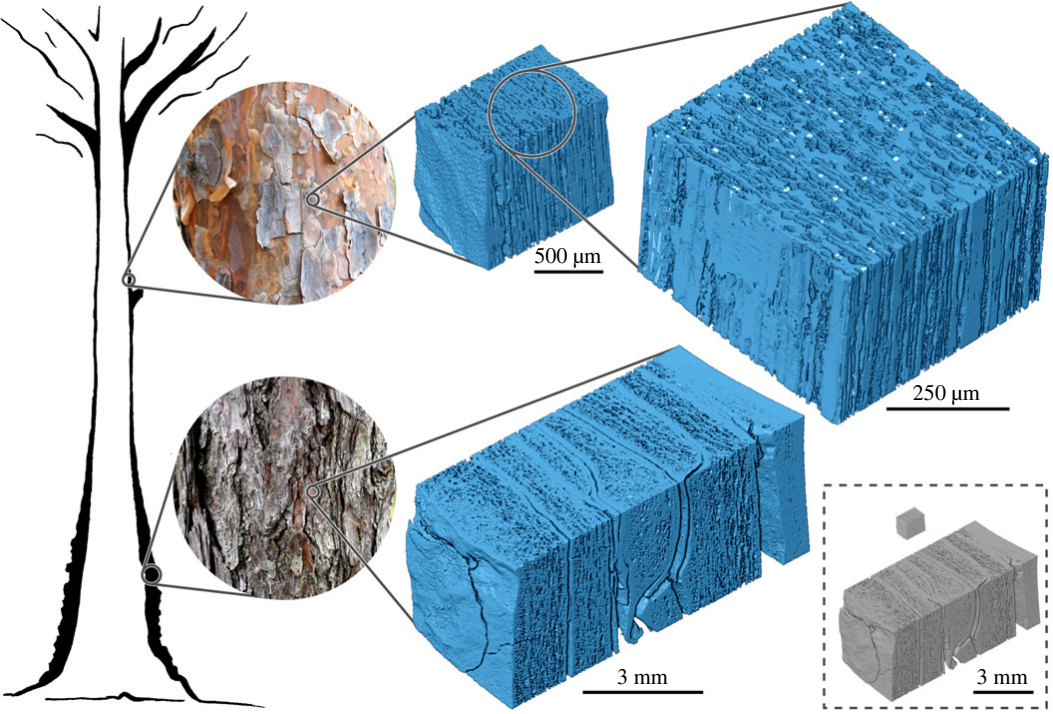
Macro- and microstructure of pine bark. Photographs show mirror bark and flaky bark from the bottom of the trunk. µCT scans of mirror bark, at high magnification low-density regions with large pores show dilated parenchyma, high-density regions are collapsed sieve tubes and periderm remnants, elongated crystal tubes appear bright, fibres are missing—a characteristic for pine bark [31]. µCT scan of flaky bark show periderm layers (dense) as well as large volume fractions of rhytidome. Grey bark cubes display size relation between flaky old bark and mirror bark. (Online version in colour.)

Changes in bark properties between tree species, within the same species and even within a tree can be highly diverse as, for example, in pine: *Pinus sylvestris*, possesses a thin so-called mirror bark when young and further up the stem and a thick, flaky bark at the bottom of older trees (figure 5).

**Figure 6.**
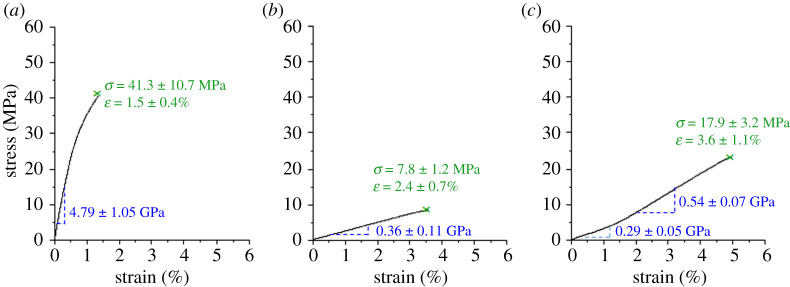
Representative stress–strain curves of dry (laboratory conditions), wet and glycerine-treated mirror bark of pine. The values show arithmetic mean values and standard deviations of 20 (*a*), 18 (*b*) and 19 (*c*) successfully tested samples. (Online version in colour.)

**Figure 7.**
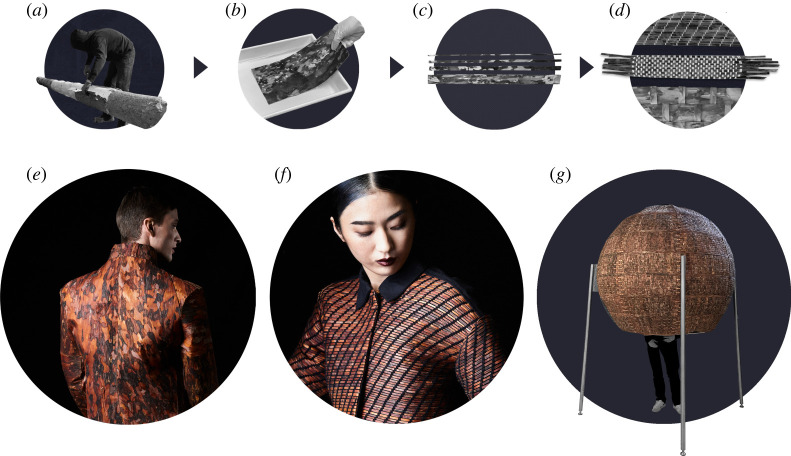
Flexible bark: (*a*) peeling a tree stem, (*b*) bark in glycerine–water solution, (*c*) bark strips, (*d*) different weaving patterns, (*e,f*) bark jackets and (*g*) walk-in bark sphere. (Online version in colour.)

The availability of bark with different structures in one tree led to the idea to explore the potential use of native pine bark in a close collaboration between science and design. Material structure and properties of bark as well as its ‘low value’ were considered to make a material that could be readily recycled, used in a biorefinery or as an energy source after its lifetime. Hence, hazardous chemicals or energy-intense processes were avoided. Like wood, bark is a large-scale material. To retrieve it from the tree, the historic method of hand-peeling, typically applied in early spring when trees are full of water, was used. With the aid of simple wedge-shaped tools, pieces of mirror bark as long as 10 m could be harvested from pine trees grown in the vicinity of Potsdam, Germany. Directly afterwards the bark is very flexible. Similar to wood, the flexibility, mechanical and swelling properties depend strongly on the moisture content, and as the bark dries, it becomes harder, stiffer and brittle causing crumpling and fracture of the mirror bark (*Pinus sylvestris*). The drying-related changes were quantified by performing tensile tests on wet, and dry bark strips with a thickness of approximately 1–2 mm, a width of 10 mm and a test length of 80 mm at a test speed of 0.04 mm s^−1^ along the fibre orientation. The experiments confirmed the observation, that dry bark is stiffer (4.7 GPa) and more brittle than wet (360 MPa) (figure 6*a,b*). To retain the bark in a flexible state, we experimented with glycerine, a method previously used to conserve leaves [74]. The peeled pine bark (figure 7*a,b*) was dried and stored under ambient conditions and was immersed in solutions of glycerine (SV Liquid Production GmbH, Glycerin 99.7% USP/EP) and water with mixing ratios of 1 : 0, 1 : 1, 1 : 2, 1 : 4, 1 : 6, 1 : 8 for 48 h. A ratio of 4 parts water and 1 part glycerine led to a flexible bark which was still dry to the touch. Glycerine-treated bark samples were also mechanically tested and showed improved flexibility and stress at failure compared to wet bark. Interestingly, the shape of the stress–strain curve changed to a bi-phasic behaviour with an initial stiffness of 290 MPa increasing to 540 MPa (figure 6*c*).

The feel and colour of the glycerine-treated (flexible) bark is similar to leather, the animal skin, tanned with substances derived from tree bark. Leather is an attractive material for the design discipline, with designers and researchers currently looking for replacement materials without animal origin [75]. Being multi-layered, and based on fibrous elements, bark could be seen as the skin of the plant, hence a structure that works as a connection to and trading zone with the environment as well as an enclosure. Humans often use leather as a kind of second skin and protection from the environment, like in clothes and shoes that protect against the cold or wetness. Even the words ‘wooden hut’ contain an old kinship with the function of skin that is remembered in language: the German word ‘Hütte’ (hut) is etymologically related to ‘Haut’ (skin), via the Sanskrit root ‘sku’ for ‘bedecken’ (to cover) [76]. However, if we want to address the design purposes of today, by using waste bark material as a resource, we need to ask: What structure–property–function relations can be translated from the bark to the realm of the human beings?

In order to create a sensation of being covered by bark, a jacket was created. Flexible mirror bark of pine was cut into pieces and sown into a jacket (figure 7*b,e*). Compared to leather, flexible tree bark is more than five times stiffer along its direction of fibre. Bark is highly anisotropic since the majority of fibrous elements run parallel to each other (µCT scans in figure 5). The high stiffness in fibre direction restricted large movements of the person wearing the jacket; across the fibre direction, the jacket was fragile. As a consequence, the model could not even lift his arms. Another limit in using bark in its pure form became apparent due to the need to extract large defect-free bark pieces for sewing, while a large proportion of the harvested bark remained as offcuts. The lack of flexibility, the barks' anisotropy and the amount of waste material were addressed by weaving two fibres or threads as rectangular interlacing, which can be used either to support or to impede each other's material characteristics [54]. Through weaving, it was possible to make use of the better mechanical properties in the fibre direction and to process narrower pieces of bark as threads (figure 7*c*). Furthermore, with different weaving patterns (figure 7*d*), it is possible that strength and flexibility can be modulated and joined without permanent incorporation of other substances such as glues.

For the woven bark jacket a twill weave, one of the basic three weave types (plain, twill, satin) [54] was used (figure 7*f*). This type of weave is used in denim and gives the cotton thread, which is tear-resistant for this purpose, a degree of flexibility that is necessary for jacket and trouser fabric. The pieces for the jacket were cut from large woven fabrics, giving and the required flexibility for movement.

Both jackets are prototypes of bark textiles. However, bark is a large-scale material and the question is if a bark textile can be used more effectively by upscaling the dimensions of application. As we mentioned, bark was also used for temporary huts. In the case of the woven bark jacket as well as in the temporary hut, barks were used at larger scales but geometries were limited to what was available. For the early huts that are mentioned by Vitruvius in the first century BC, techniques of weaving were used and thus rudiments of geometric arrangements [38]. This of course in a simple form, when branches with leaves were woven into a rough framework of branches to create a roof or walls. But also more developed techniques are known, e.g. when mats or fabrics are woven and used as wall coverings, such as in the famous ‘Caribbean hut’ that Gottfried Semper saw in the nineteenth century and documented in his theory of architecture [77]. The above-mentioned Austrian temporary huts made of bark were designed to serve two functions: to provide shelter for some time and to blend into the environment; however, these coverings were notwoven and, therefore, represent a different technique. The desire to create an object that integrates into the forest and is clearly human-made and not restricted to a certain geometry, determined the idea of a walk-in sphere made of tree bark (figure 7*g*). The use of tree bark in the form of a woven structure gives the possibility to use the protective function of the bark and to strengthen it by using different weaving patterns and thereby to increase the variety of shapes. The form of the sphere was selected to explore the limits of formability and to leave room for the viewer's own interpretation.

The use of bark fabrics for open concept spaces is intended to promote further fields of application in the future. Thus, other forms of space such as huts, pavilions, tents or indoor textiles are conceivable. The use of variable sized bark strips combined with different weaving patterns gives the possibility to design applications appropriately. This includes design concepts for end-of-life reuse of ‘waste’ materials in entirely different applications, involving just a small adaptation rather than complete reprocessing of the material.

Pine bark, larch and spruce barks were treated with glycerine but with limited success. The reasons why it works only for pine bark remain unclear. It is possible that a thicker rhythidome, and changes in cellulose orientation or chemistry are the reasons. Unfortunately, detailed knowledge about the material structure and properties of bark are scarce and/or missing and future research should address these questions as well as questions related to technological challenges, the smell, hydrophobicity, drying with time, durability or questions related to the origin of increased flexibility. Towards a more efficient and sustainable tree material use, basic knowledge about material structure and composition from the nano- to the macroscale is needed as well as relating it to functions for the tree. This will help in understanding and predicting variabilities of material properties. Further collaborations between the humanities, design and science disciplines have the potential to point us towards creative new material concepts and the design experiments themselves provide a first basic idea about the material and direct us towards targeted scientific explorations.

## 5. Summary and outlook

Trees adapt to changing conditions by growth and add material with diverse properties depending on needs. The resulting variability of tree materials complicates its use and considerable numbers of trees are currently used directly as fuel or as feedstocks in biorefineries. In times of limited resources, it would be desirable to find applications for these materials. Ideally, these materials should be used in as simple a form as possible, with limited modifications and low energy consumption in order to allow ease of recycling and use as fuel at the end of the product's lifetime.

Interdisciplinary collaborations beyond the natural sciences are helpful in the development of new sustainable material concepts. Not only does the material need to be understood, for example in the classical structure–function analysis done in biological materials science, but input from design and the humanities help to find applications and acceptance in the use of these materials. We show this with flexible bark textiles, which emerge by combining material sciences approaches with design experiments, historic knowledge and processes as well as more recent research on cellulose-based materials. We currently face limitations by our lack of knowledge of tree-based raw materials that are not straight grown tree trunks or from well-defined industrial wood of economically important species. To close the knowledge gap, research is needed to better understand tree material in a broader sense (e.g. branches and bark). A possibility to accelerate this type of research is the consideration of tacit knowledge of craftspeople who are familiar with making wooden artefacts of various kinds. By investigating the potential of this one example of bark as a textile, we hope to inspire more explorations into other waste plant materials as sustainable resources.

The understanding of plant material characteristics in relationship with the function for various tree species is a solid basis to be used as a raw material by creative designers for applications that fit its natural properties and might be combined with already existing knowledge. Indeed, modern numerical planning tools require quantitative data that are normally available only for generic materials types, such as spruce wood from the straight part of the stem (figure 1), for example. The vision is that quantitative knowledge of the properties of a specific piece of material will allow designers to engage like craftspeople with this piece, but using all the modern numerical planning tools. This could become a revolution for the use of increasingly precious raw materials, similar to the breakthrough of personalized medicine in healthcare.

## Footnotes

1 All examples are taken from the collection of the British Museum. See museum numbers Oc.4061 (netted bag made of bark fibre cord, made by aboriginal Australian, nineteenth century), 2016,8030.1 (bag made of plaited strips of birch bark from Karelia in Russia/former Finland, 1900–1940), Am1928,-.92 (mat made of birch bark, with quill works and feathers, from North America, bought 1928), 2011,8036.35 (pot scourer in the form of a ball, made of curled birch bark strips, from Finland, 2011), Am1949,22.170 (scroll made of birch bark, with zoomorphic drawings, made by the Ojibwa, Minnesota, bought 1949), Oc1894,0814.4 (Oblong sheet of barkcloth with geometrical designs from Taveuni in Fiji/Oceania, nineteenth century). www.britishmuseum.org/collection (last accessed 6 March 2021).

2 The manuscripts were found at the beginning of the 1990s and came to the British Museum in 1994. See Or. 14195.17, 19–21, 27–27, 35. www.britishmuseum.org/collection (last accessed 6 March 2021).

## Data accessibility

The tensile test data and µCT scans (radiographies and reconstructed slices, including all metadata) are available from edmond—the Open Research Data Repository of the Max Planck Society (https://edmond.mpdl.mpg.de/imeji/collection/4oVoMRlV1tcHcLZI/share?q=&fq=).
